# Re-expansion pulmonary edema following a pneumothorax drainage in a patient with COVID-19

**DOI:** 10.1186/s12890-021-01661-w

**Published:** 2021-09-16

**Authors:** Kosaku Komiya, Ryosuke Hamanaka, Hisayuki Shuto, Hiroki Yoshikawa, Atsushi Yokoyama, Kazufumi Hiramatsu, Jun-ichi Kadota

**Affiliations:** Respiratory Medicine and Infectious Diseases, Faculty of Medicine, Oita University, 1-1 Idaigaoka, Hasama-machi, Yufu, Oita 879-5593 Japan

**Keywords:** Re-expansion pulmonary edema, Pneumothorax, COVID-19

## Abstract

**Background:**

Re-expansion pulmonary edema is an uncommon complication following drainage of a pneumothorax or pleural effusion. While pneumothorax is noted to complicate COVID-19 patients, no case of COVID-19 developing re-expansion pulmonary edema has been reported.

**Case representation:**

A man in his early 40 s without a smoking history and underlying pulmonary diseases suddenly complained of left chest pain with dyspnea 1 day after being diagnosed with COVID-19. Chest X-ray revealed pneumothorax in the left lung field, and a chest tube was inserted into the intrathoracic space without negative pressure 9 h after the onset of chest pain, resulting in the disappearance of respiratory symptoms; however, 2 h thereafter, dyspnea recurred with lower oxygenation status. Chest X-ray revealed improvement of collapse but extensive infiltration in the expanded lung. Therefore, the patient was diagnosed with re-expansion pulmonary edema, and his dyspnea and oxygenation status gradually improved without any intervention, such as steroid administration. Abnormal lung images also gradually improved within several days.

**Conclusions:**

This case highlights the rare presentation of re-expansion pulmonary edema following pneumothorax drainage in a patient with COVID-19, which recovered without requiring treatment for viral pneumonia. Differentiating re-expansion pulmonary edema from viral pneumonia is crucial to prevent unnecessary medication for COVID-19 pneumonia and pneumothorax.

## Background

Re-expansion pulmonary edema following pneumothorax or pleural effusion drainage has already been reported for decades, with an incidence of approximately 1% [1]. Although its pathophysiology remains poorly understood, re-expansion pulmonary edema is believed to be caused by increased permeability of the pulmonary capillaries through inflammatory reactions. Re-expansion and reperfusion of a previously collapsed lung tissue may cause inflammations with reactive oxygen species and superoxide radicals and thereby may increase the capillary permeability [2, 3]. For other mechanisms, increased pulmonary hydrostatic pressure caused by venous return, pressure-induced mechanical disruption of the alveolar capillaries and decreased functional surfactant levels may also be suggested to result in re-expansion pulmonary edema [4].

Pneumothorax has been reported as a complication in patients with COVID-19, requiring hospital admission, and may be associated with increased mortality [5, 6]. A recent large observational study conducted in the United Kingdom revealed that 1283 (0.97%) of 131,679 patients aged > 18 years, who were admitted to hospitals for COVID-19, had pneumothorax at some stages during their admission [7]. The incidence differed between groups according to the level of respiratory support: 0.16% of patients not requiring supplemental oxygen, 0.56% of patients requiring oxygen without pressure support, 0.96% of patients treated with non-invasive respiratory support and 6.1% of patients who received invasive ventilation for pneumothorax. Multivariate analysis in this study showed that male sex, smoking, chronic pulmonary disease and invasive ventilation were associated with increased risk of pneumothorax.

Considering that approximately 2 billion people have already been infected with severe acute respiratory syndrome coronavirus 2 (SARS-CoV-2) worldwide, a certain number of patients with COVID-19 and pneumothorax should have suffered from re-expansion pulmonary edema following a drainage procedure. However, no such case has been reported. Herein, we present the case of a patient with COVID-19 diagnosed with re-expansion pulmonary edema following pneumothorax drainage and discuss the importance of differentiating re-expansion pulmonary edema from COVID-19 pneumonia.

## Case presentation

**Fig. 1:**
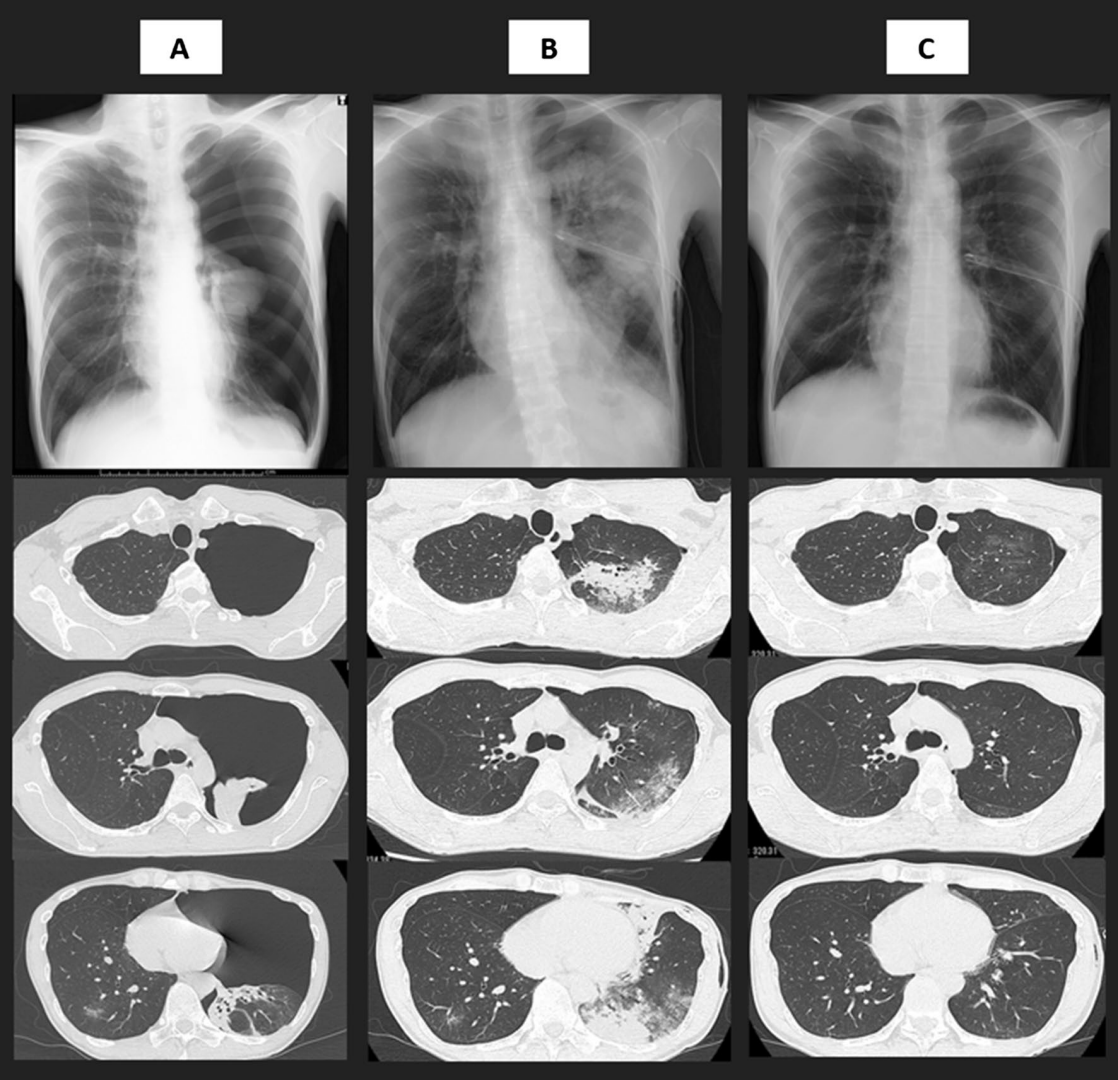
Chest X-ray and thin-section computed tomography at upper lobes, carina and lower lobes levels on admission (**A**), 1 day after thoracic drainage procedure (**B**) and 7 days after thoracic drainage procedure (**C**)

A man in his early 40s had a low-grade fever and was positive for SARS-CoV-2 in the polymerase chain reaction result 5 days after. He did not have a smoking history and underlying pulmonary diseases. Due to no symptoms except for the low-grade fever, chest X-ray had not been performed at diagnosis of COVID-19. However, 1 day after diagnosis, he suddenly complained of left chest pain with dyspnea and visited our hospital. Physical examination revealed a body temperature of 37.7 °C, an oxygen saturation (SpO_2_) of 93% without supplemental oxygenation, blood pressure of 117/81 mmHg and heart rate of 99 beats/min. Laboratory tests revealed a normal leukocyte count (7320 cells/μL) and slightly elevated serum C-reactive protein levels (0.30 mg/dL). Chest X-ray revealed significant pneumothorax in the left lung field (Fig. 1A), with 78% of the Light index [8]. Chest computed tomography (CT) performed to determine appropriate thoracic space for drainage tube revealed left lung collapse without pneumomediastinum and patchy ground-glass opacity (GGO) in the right lower lobe. We inserted a chest drainage tube (20 Fr) into the intrathoracic space without negative pressure 9 h after he reported chest pain and his respiratory symptoms disappeared and SpO_2_ improved to 98%; however, after 2 h, he re-experienced dyspnea with a lower SpO_2_ of 92%. Chest X-ray revealed an improved collapse but infiltration of the expanded lung. Chest CT performed on the following day to distinguish re-expansion pulmonary edema from COVID-19 pneumonia revealed extensive consolidation with ground-glass opacities in all lobes of the expanded left lung (Fig. 1B). No significant morphological abnormality as a risk for pneumothorax has been observed. Re-expansion pulmonary edema was diagnosed instead of COVID-19 pneumonia progression based on this clinical course. His dyspnea and oxygenation status gradually improved without any intervention, including supplemental oxygen, diuretics or steroid administration, and abnormal lung involvements observed in the chest CT were diminished 7 days after the chest drainage procedure (Fig. 1C).

## Discussion and conclusions

Herein, we present a case of re-expansion pulmonary edema following pneumothorax drainage with COVID-19. No significant lung abnormality including bullae has been observed for the risk for pneumothorax in chest CT. SARS-CoV-2 infection might have been a trigger for pneumothorax, but no clear evidence was obtained in this case, although pneumonia was not extensive.

First, whether abnormal involvements in the left lung represent re-expansion pulmonary edema or COVID-19 pneumonia needs further investigation. Symptoms of re-expansion pulmonary edema include chest discomfort, persistent severe cough, production of frothy sputum and dyspnea, which are commonly observed in patients with COVID-19. However, these symptoms have been reported to start within 1–2 h in 64% of patients with re-expansion pulmonary edema [4]. Dyspnea with decreased oxygenation status occurred 2 h after a drainage procedure, which is clinically compatible with re-expansion pulmonary edema. Furthermore, radiological features in the left lung after expansion do not appear to be COVID-19 pneumonia. Typical CT findings of COVID-19 pneumonia are predominantly peripheral patchy GGOs and/or consolidations, and vascular enlargement is commonly reported [9]. In this patient, consolidation was predominantly observed in the central field of the left upper lobe without vascular enlargement, which may support hydrostatic edema [10], whereas a patchy GGO in the right lower lobe is consistent with COVID-19 pneumonia. Moreover, the extensive consolidation with GGO has almost disappeared within several days, which is in agreement with the characteristics of re-expansion pulmonary edema. As the majority of patients with re-expansion pulmonary edema completely recover within 5–7 days [3], the extent of lung abnormalities caused by COVID-19 peaks at days 6–11 and gradually disappears [11]. The course of rapid improvement also supports that abnormal images were caused by re-expansion pulmonary edema.

Re-expansion pulmonary edema following a pneumothorax or pleural effusion drainage rarely occurs [1]. Proposed risk factors include collapse duration of > 72 h and the application of high negative pressures during thoracic drainage (> 20 cm H_2_O) [4]. Although these risk factors were not observed in the present case, he suffered from re-expansion pulmonary edema. In a small retrospective study [12], pneumothorax size was associated with the occurrence of re-expansion pulmonary edema. The large size of pneumothorax in the current case might have accelerated the development of re-expansion pulmonary edema. The SARS-CoV-2 infection could be associated with an increased risk for re-expansion pulmonary edema. SARS-CoV-2 infection is known to cause cytokine-mediated endothelialitis in the pulmonary vascular system and increases transepithelial permeability [13]. Hypoxia also decreases active sodium transport across the alveolar wall and reduces alveolar fluid reabsorption [14]. In an acid aspiration lung injury mouse model, knockout of angiotensin-converting enzyme 2 (ACE2), a known main receptor of SARS-CoV-2, has been reported to worsen pulmonary edema [15]. Indeed, COVID-19 infection causes a functional ACE2 deficiency in the lung and increases the production of proinflammatory cytokines and extends half-time of some cytokines including bradykinin [16]. Moreover, COVID-19 is characterized by an increased incidence of pulmonary embolism [17]. This phenomenon increases the capillary hydrostatic pressure and pulmonary vascular resistance, as well as precapillary and postcapillary resistance [18], which may result in the development of pulmonary edema. However, no evidence has been established on whether the risk of re-expansion pulmonary edema is related to the severity of COVID-19 pneumonia. At least, physicians need to be aware of general risk factors for re-expansion pulmonary edema including long duration of collapse, pneumothorax size and high negative pressure for drainage even in a patient with COVID-19 and pneumothorax.

Whether re-expansion pulmonary edema following thoracic drainage is more prone to occur in patients with COVID-19 than that in the general population, the incidence would be underestimated because no case of re-expansion pulmonary edema with COVID-19 has been reported. Since pneumothorax is more likely to occur in patients with severe COVID-19 pneumonia requiring invasive ventilation, distinguishing re-expansion pulmonary edema from extensive COVID-19 pneumonia would be challenging. However, if re-expansion pulmonary edema is misdiagnosed as progressive COVID-19 pneumonia and then escalated medication is administered including additional corticosteroid, it would worsen the prognosis of pneumothorax. Differentiating possibly underestimated re-expansion pulmonary edema following thoracic drainage from COVID-19 pneumonia could effectively improve the disease prognosis.

## Data Availability

Data are available in this manuscript.

## Abbreviations

ACE2: Angiotensin-converting enzyme 2
COVID-19: Coronavirus disease 2019
CT: Computed tomography
GGO: Ground-glass opacity
SARS-CoV-2: Severe acute respiratory syndrome coronavirus 2
